# Differences in human immunodeficiency virus-1C viral load and drug resistance mutation between plasma and cerebrospinal fluid in patients with human immunodeficiency virus-associated cryptococcal meningitis in Botswana

**DOI:** 10.1097/MD.0000000000022606

**Published:** 2020-10-09

**Authors:** Nametso Kelentse, Sikhulile Moyo, Mompati Mogwele, Kwana Lechiile, Natasha O. Moraka, Dorcas Maruapula, Kaelo K. Seatla, Lerato Esele, Kesaobaka Molebatsi, Tshepo B. Leeme, David S. Lawrence, Rosemary Musonda, Ishmael Kasvosve, Thomas S. Harrison, Joseph N. Jarvis, Simani Gaseitsiwe

**Affiliations:** a Botswana Harvard AIDS Institute Partnership; b University of Botswana, Department of Medical Laboratory Sciences, Gaborone, Botswana; c Harvard T.H. Chan School of Public Health, Department of Immunology and Infectious Diseases, Boston, United States; d Stellenbosch University, Department of Pathology, Stellenbosch, South Africa; e Botswana-University of Pennsylvania Partnership, Gaborone, Botswana; f Department of Clinical Research, Faculty of Infectious and Tropical Diseases, The London School of Hygiene and Tropical Medicine, London, United Kingdom; g University of Botswana, Department of Statistics, Gaborone, Botswana; h Centre for Global Health, Institute for Infection and Immunity, St. George's University of London, United Kingdom; i University of Botswana, Department of Biological Sciences, Gaborone, Botswana; j Division of Infectious Diseases, Department of Medicine, Perelman School of Medicine, University of Pennsylvania, Philadelphia, PA, USA.

**Keywords:** cerebrospinal fluid, compartmentalisation, cryptococcal meningitis, drug resistance mutations, human immunodeficiency virus, viral escape

## Abstract

To determine effects of cryptococcal meningitis (CM) on human immunodeficiency virus (HIV)-1C cerebrospinal fluid (CSF) viral escape, CSF/plasma viral discordance, and drug resistance mutation (DRM) discordance between CSF and plasma compartments, we compared CSF and plasma viral load (VL) and DRMs in individuals with HIV-associated CM in Botswana.

This cross-sectional study utilized 45 paired CSF/plasma samples from participants in a CM treatment trial (2014–2016). HIV-1 VL was determined and HIV-1 protease and reverse transcriptase genotyping performed. DRMs were determined using the Stanford HIV database. CSF viral escape was defined as HIV-1 ribonucleic acid ≥0.5 log_10_ higher in CSF than plasma and VL discordance as CSF VL > plasma VL.

HIV-1 VL was successfully measured in 39/45 pairs, with insufficient sample volume in 6; 34/39 (87.2%) participants had detectable HIV-1 in plasma and CSF, median 5.1 (interquartile range: 4.7–5.7) and 4.6 (interquartile range:3.7–4.9) log_10_ copies/mL, respectively (*P*≤.001). CSF viral escape was present in 1/34 (2.9%) and VL discordance in 6/34 (17.6%). Discordance was not associated with CD4 count, antiretroviral status, fungal burden, CSF lymphocyte percentage nor mental status. Twenty-six of 45 (57.8%) CSF/plasma pairs were successfully sequenced. HIV-1 DRM discordance was found in 3/26 (11.5%); 1 had I84IT and another had M46MI in CSF only. The third had K101E in plasma and V106 M in CSF.

Our findings suggest that HIV-1 escape and DRM discordance may occur at lower rates in participants with advanced HIV-disease and CM compared to those with HIV associated neurocognitive impairment.

## Abstract 2

Supplemental Digital Content is available in the text

## 1 Introduction

Successful roll-out of antiretroviral therapy (ART) has significantly reduced morbidity, mortality and incidence of opportunistic infections associated with Human Immunodeficiency Virus (HIV) infection and acquired immune deficiency syndrome.^[1]^ However, Sub-Saharan Africa continues to be disproportionately burdened by the HIV-1 epidemic and the region has the greatest number of deaths, morbidity, and opportunistic infections.^[2]^ One of the major biological barriers to complete eradication of HIV-1 is the ability of the virus to compartmentalise in reservoir sites such as the central nervous system (CNS), lymphoid tissue, gut tissues, and other anatomical sites.^[3]^ The CNS can serve as a sanctuary for HIV-1 replication resulting in persistent infection, emergence of drug resistance mutations (DRM), and the development of HIV-associated neurocognitive disorders.^[4]^

After HIV-1 seeds to the CNS, compartmentalisation and replication in the CNS may yield elevated cerebrospinal fluid (CSF) HIV-1 ribonucleic acid (RNA) despite undetectable or low viral loads (VL) in the plasma, a phenomena commonly known as CSF viral escape or discordance.^[5,6]^ The global prevalence of CSF viral escape is reported to be between 0.7% and 27.4%.^[7]^ Factors such as low nadir CD4 count, ART treatment interruptions, persistent low level viremia and poor penetration of some antiretroviral drugs (ARV) through the blood brain barrier (BBB) into the CNS contribute to CSF viral escape.^[8,9]^ In addition, discordant DRMs in the CSF and plasma can influence the development of CSF HIV-1 viral escape and limit future effectiveness of ARVs.^[5,10]^ Furthermore, sub-optimal penetration by some ARVs into certain anatomical sites can lead to decreased therapeutic drug levels in the CSF thus promoting development of DRMs.^[11]^

One of the major causes of acquired immune deficiency syndrome-related morbidity and mortality is HIV-associated cryptococcal meningitis (CM).^[12]^ There are still conflicting data relating to effects of CM on CSF HIV-1 VL. It has been suggested that CM causes an inflammation of the meninges that allows leucocytes harbouring HIV-1 to enter CNS leading to an increased HIV-1 VL.^[13]^ However, a direct association between high CM burden and high HIV-1 VL has not been shown.^[13,14]^ Furthermore, there are no data regarding discordant DRMs in the context of HIV-associated CM, which may be of clinical and public health relevance given the increasing proportion of CM patients who are now ART-experienced.^[15,16]^

To address these knowledge gaps, the aim of this study was to investigate HIV-1C CSF viral escape and VL discordance as well as DRM discordance between CSF and plasma compartments among people living with HIV-associated CM.

## 2 Materials and methods

### 2.1 Study design

A retrospective cross-sectional study utilizing archived samples and existing patient data from the “AMBIsome Therapy Induction OptimisatioN for Cryptococcal Meningitis” (AMBITION-cm) study was performed. AMBITION-cm was a multi-site phase-II non-inferiority trial which aimed to investigate the early fungicidal activity of 3 short-course, high-dose liposomal amphotericin regimens for CM. The trial recruited 80 participants across 2 sites: Princess Marina Hospital (Gaborone, Botswana) and Bugando Medical Centre and Seko Toure Hospital (Mwanza, Tanzania) between October 2014 and September 2016.^[16]^

### 2.2 Study population

We tested archived CSF and plasma samples from AMBITION-cm participants recruited in Gaborone, Botswana. All AMBITION-cm participants were confirmed to have HIV by a rapid diagnostic test prior to enrolment or evidence of a previously documented positive test or detectable HIV-1 VL. CM infection was determined by positive India Ink or cryptococcal antigen in CSF. We used samples collected on study day 3 or 7 depending on their availability; 45 participants had paired CSF and plasma samples available and were subsequently included in this study.

### 2.3 Ethics

The study was approved by the University of Botswana Institutional Review Board and a research permit was obtained from Botswana Ministry of Health and Wellness (HPDME:13/18/1). All participants had previously consented to the use of their stored samples in future research into the pathophysiology of HIV-associated CM in the parent study (ISRCTN#10248064).

### 2.4 Laboratory investigations

#### 2.4.1 HIV-1 Viral Load Assay

CSF samples were centrifuged at 1200xg for 10 minutes in order to obtain cell-free supernatant. HIV-1 VL was measured in plasma and CSF using Abbott m2000rt/m2000sp assay (Abbott Laboratories, Abbott Park, Illinois, U.S.A). Samples with volumes below 800 μL were diluted with phosphate buffered saline in a 1:5 ratio and the resulting VL multiplied by the dilution factor as per manufacturer's instructions. CSF viral escape was defined as HIV-1 VL ≥ 0.5log_10_ copies/mL higher in CSF than plasma VL while CSF/Plasma HIV-1 VL discordance was defined as any CSF HIV-1 VL greater than plasma VL as previously described.^[5,17]^

#### 2.4.2 RNA extraction

HIV-1 RNA was extracted from 140 μL of plasma and CSF supernatant using QIAamp RNA extraction mini kit (Qiagen, Hilden, Germany) according to the manufacturer's instructions.

### 2.5 Amplification of the *pol* gene

Complementary DNA from protease and reverse transcriptase (RT) of the HIV-1 *pol* gene was synthesized in a one-step polymerase chain reaction (PCR) using transcriptor 1-step PCR kit (Roche Applied Science, Germany) as previously described.^[18]^

Phusion High Fidelity DNA Polymerase PCR protocol (New England Biolabs, Hitchin, UK) was used for second round PCR according to the manufacturer's instructions. The primers used contained equal volumes of 2 μM forward (LNAF1; 5’-GAAGGACCAAATGAAAGAYTG-3’) and 2.5 μM reverse primer (RT20C; 5’-CTGCCAATTCTAACTGCTTC-3’). Second round amplicons were loaded on a 1% agarose gel in Tris-Borate- ethylenediaminetetraacetic acid (TBE) buffer and run at 100 V for 45 minutes in order to check amplification success.

### 2.6 Sanger sequencing

Following purification of the second-round amplicons, sequencing PCR was done using Big Dye Terminator sequencing kit according to the manufacturer's protocol (Applied Biosystems, Foster City, CA). The 7 primers used for sequencing were as previously described.^[18]^ These were designed such that atleast 2 primers overlap at any given position of the amplicon. The sequences generated were then purified using ZR DNA sequencing clean-up kit (Zymo Research, CA) then determined on the ABI Prism 3130xl genetic analyzer (Applied Biosystems, Foster City, CA).

### 2.7 Phylogenetic analyses

Raw sequences were edited manually using Sequencher software version 5.0 (Gene codes Corporation, Ann Arbor, MI). To identify HIV-1 DRMs, the consensus sequences were analysed in the Stanford HIV Drug Resistance Database. The consensus sequences were aligned with HIV-1 HXB2 and subtype reference sequences from Los Alamos National Laboratory HIV database in Bioedit version 7 software. Neighbour-joining phylogenetic tree was constructed in MEGA version 7 software with 1000 bootstrap replicates to check for contamination and relatedness (Substitution model: Tamura-2-parameter, T92+G+I). All HIV-1 sequences described in this study were submitted to Genbank (accession numbers: MT663164-MT663215).

### 2.8 Statistical analysis

Wilcoxon signed rank test was used to compare paired CSF and plasma HIV-1 VL. Spearman rank correlation was used to assess the correlation between CSF and plasma VL. Fisher's exact test or logistic regression was used to analyse categorical data where appropriate. Factors associated with CSF/ plasma HIV-1 VL discordance and mental status were determined using binary logistic regression. To adjust for confounders, multivariable analysis with covariates selected a priori was performed. *P*-values < .05 were considered to be statistically significant. All analyses were performed using R version 3.6.0.^[19]^

## 3 Results

### 3.1 Participants characteristics

The participants enrolled in the AMBITION-cm study in Botswana from whom samples were obtained were predominantly male 28/45 (62.2%) and the median age was 38 (Q1, Q3: 32–44) years. The baseline median CD4+ T-cell count was 28 (Q1, Q3: 10–46) cells/uL and 12/45 (26.7%) participants were on ART, mostly Tenofovir Disoproxil Fumarate, Emtricitabine and Efavirenz (tenofovir disoproxil fumarate/ emtricitabine / efavirenz) regimen. A total of 13/45 (28.8%) participants had an abnormal mental status at baseline and the overall median CSF fungal burden as determined by quantitative cryptococcal culture was 5.0 (Q1, Q3: 3.7–5.6) log_10_ cells/mL (Table 1).

**Table 1:**
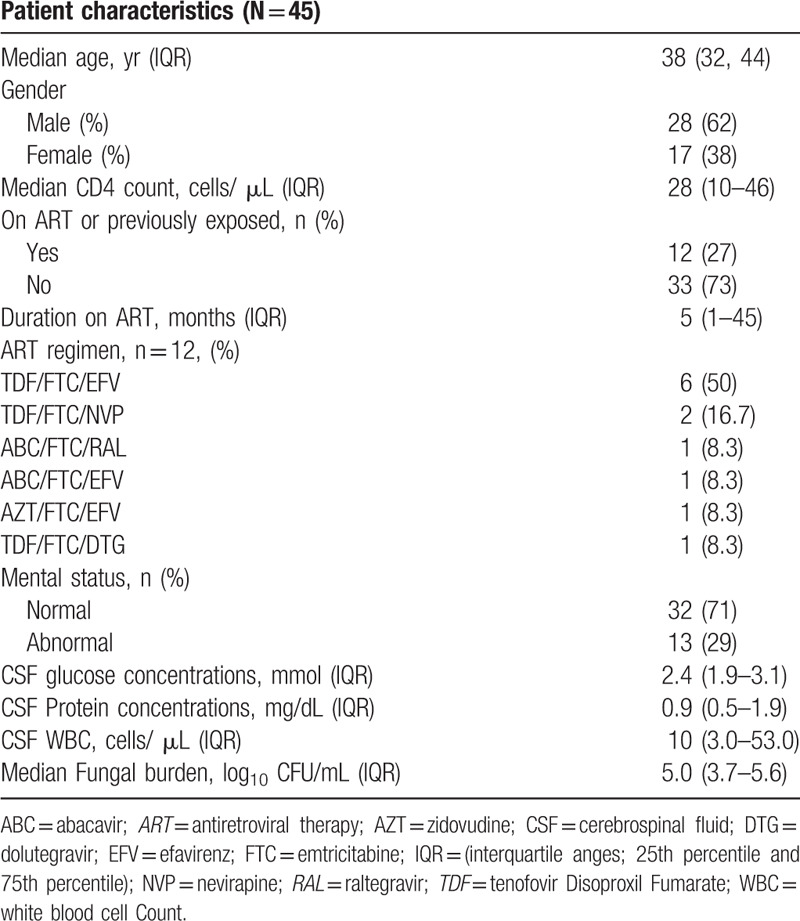
Baseline characteristics and demographics of the 45 study participants.

### 3.2 Relationship between CSF and Plasma HIV-1 VL

**Figure 1:**
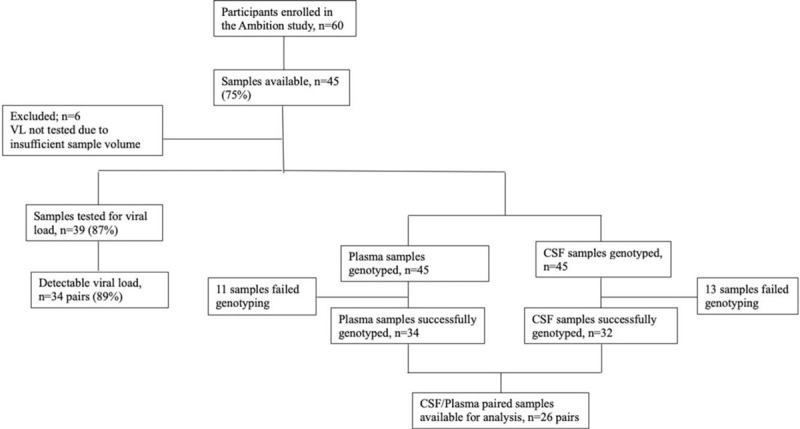
Consort diagram showing enrolment criteria, study design and study population.

**Figure 2:**
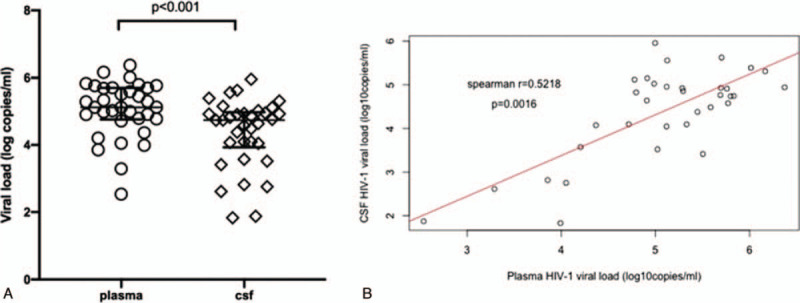
(A) Comparison of median HIV–1 viral load in plasma and Cerebrospinal fluid (CSF) paired samples, n = 34. (B) The relationship between Cerebrospinal fluid (CSF) and plasma HIV-1 viral load, n = 34.

Among 45 CSF/plasma paired samples available, 39 pairs were tested for VL and 6 pairs were excluded due to insufficient volumes (Fig. 1). A total of 34/39 participants (87.2%) had detectable HIV-1 VL in plasma and CSF with medians of 5.1 (interquartile range: 4.8–5.7) and 4.6 (interquartile range: 3.8–4.9) log_10_ copies/mL, respectively (*P* < .001) (Fig. 2A). There was a weak positive linear relationship between the CSF and plasma HIV-1 VL (Spearman *r* = .5218, *P* = .002) (Fig. 2B).

### 3.3 CSF viral escape and CSF HIV-1 VL discordance

CSF HIV-1 viral escape was observed in 1/34 (2.9%) [95% CI: 0.07–15.30]. The participant was ART naïve with median HIV-1 VL of 5.0 (5.0–5.0) and 6.0 (6.0–6.0) log_10_ copies/mL in plasma and CSF, respectively. This individual had an abnormal mental status and died before the end of the trial.

HIV-1 VL discordance was observed in 6/34 (17.6%) pairs. Discordance was not associated with CD4+ T-cell count, protein and glucose concentration, age, fungal burden, CSF lymphocytes percentage or ART status (Table 2). No correlation was observed between CSF HIV-1 VL and fungal burden (Spearman *r* = 0.06, *P* = .71, protein concentration (Spearman *r* = 0.16, *P* = .35), lymphocyte percentage (Spearman *r* = -0.06, *P*= .80) or CD4+ T-cell count (Spearman *r* = 0.07, *P* = .67).

**Table 2:**
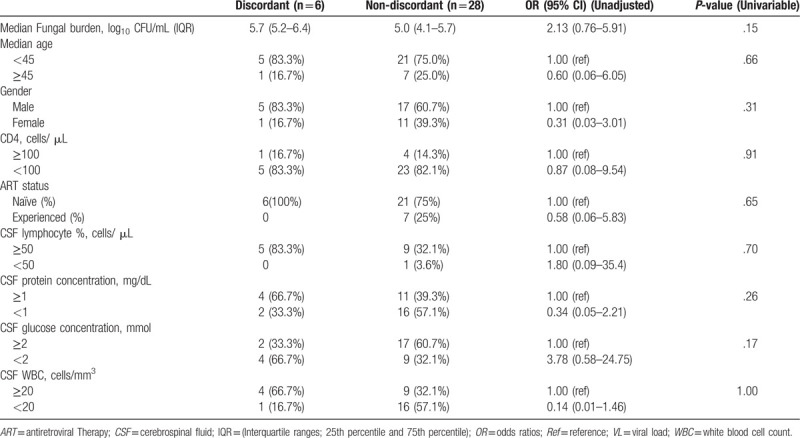
Factors associated Cerebrospinal Fluid and plasma HIV-1 VL discordance.

### 3.4 Association between CSF/Plasma HIV-1 VL discordance and mental status

Participants who had HIV-1 VL discordance had 7-fold higher odds of abnormal mental status than those who were non-discordant (*P* = .04). However, after adjusting for fungal burden, CSF HIV-1 VL, ART status and CD4+ T-cell count, this did not reach statistical significance (adjusted odds ratio (aOR) = 7.88, 95% confidence intervals (CI) = 0.75–83.4, *P* = .09) (See Table 1, Supplemental content 1, http://links.lww.com/MD/E974, which illustrates predictors of mental status in participants with HIV-associated Cryptococcal Meningitis). Median CSF HIV-1 VL was higher in participants with abnormal mental status than those with normal mental status reaching borderline statistically significance (*P* = .05). However, CSF HIV-1 VL and ART status were not predictors of mental status in the adjusted analysis (aOR = 2.07 per log_10_ increase in VL, 95% CI = 0.49–8.68, *P* = .32; and on ART aOR = 1.56, 95% CI = 0.07–37.5, *P* = .78).

### 3.5 HIV-1 *Pol* drug resistance associated mutations

All the 45 participants had CSF/plasma paired samples available for genotyping and 34 plasma and 32 CSF samples were successfully genotyped. However, only 26 were paired CSF/plasma samples (See Figure, Supplemental content 2, http://links.lww.com/MD/E978, which shows a Neighbour-joining phylogenetic tree generated from HIV-1C protease and RT sequences of 26 CSF and plasma paired samples.). Of the 26 pairs, 5 participants had HIV-1 strains with DRMs. The most predominant mutation was K101E, occurring in HIV-1 strains of 2 plasma samples and in 1 CSF sample. All other mutations occurred at an equal frequency of 1 in plasma and CSF (Table 3). HIV-1 DRM discordance was present in 3/26 (12%) HIV-1 strains of the paired samples. Of these, one had HIV-1 strain harbouring I84IT and the other had M46MI protease inhibitor (PI)-associated mutation in CSF but not in the plasma. The third participant had HIV-1 strain with RT mutation K101E in plasma and V106 M in CSF.

**Table 3:**
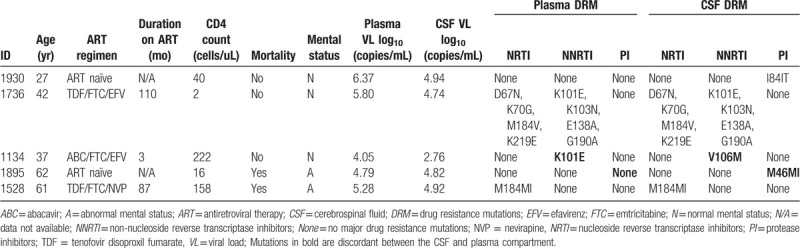
Characteristics of participants with HIV-1 Protease and Reverse transcriptase associated mutations in cerebrospinal fluid and plasma.

## 4 Discussion

In this cross-sectional study evaluating HIV-1 VL and DRM discordance between CSF and plasma in participants with HIV-associated CM, we identified only 1 case (2.9%) of CSF viral escape. We reported 17.6% of cases to have CSF/plasma HIV-1 VL discordance. HIV-1 VL was significantly higher in plasma than CSF and a weak positive linear relationship was observed between the 2 compartments. Those with CSF/plasma HIV-1 VL discordance had higher odds of abnormal mental status, although this did not reach statistical significance, and with only 1 case of CSF viral escape, we could not assess whether mental status was associated CSF viral escape.

HIV-1 VL discordance and CSF HIV-1 VL was not associated with either CD4+ T-cell count, CSF protein and glucose concentration, CSF lymphocytes percentage, CSF white cell count or fungal burden in our patient population. Increased CSF leucocyte counts and lower peripheral CD4+ T cell counts have previously been found to correlate with CSF HIV-1 viral burden and escape in participants with either neurocognitive disorders or CM presumably as HIV-1 may cross the BBB via infected cells contributing to higher HIV-1 VL in the CNS.^[13,20–24]^ A lack of correlation between CSF lymphocyte count and CSF HIV-1 VL may suggest that CNS occurs as a distinct compartment from peripheral blood and can support HIV replication or that replication occurs in different cell types in the CNS.^[24]^

A lack of association between CSF/plasma HIV-1 VL discordance, CSF HIV-1 VL and CM fungal burden in our study corroborate findings from other studies which reported that high CM burden does not contribute to elevated CSF HIV-1 VL.^[13,14]^ Some studies have reported absence of CSF viral escape at the time of CM and neurological impairment and 23.1% HIV-1 VL discordance.^[14]^ This may suggest that HIV-associated CM does not necessarily predispose to CSF viral escape. We could not assess the impact of ART exposure on HIV-1 VL discordance as all participants with HIV-1 VL discordance were ART naïve.

In a univariable analysis model, participants with HIV-1 VL discordance had 7-fold higher odds of abnormal mental status. However, after adjusting for fungal burden, CSF HIV-1 VL, ART status and CD4+ T-cell count, the relationship was not statistically significant, perhaps reflecting our small sample size. Studies show that CSF/plasma HIV-1 discordance or escape is a predictor of neurocognitive disorder, however, these studies have evaluated effects of CSF viral escape on progressive neurocognitive disorders whereas our study focused mainly on pathogenic neurological infection in the CNS and the relatively low number of participants with discordance, may have overshadowed our ability to determine the impact of a slightly higher CSF HIV-1 VL on cognitive function.^[17,25]^

Among 5 participants harbouring drug resistant HIV-1 strains, 3 had DRM discordance. In 1 participant, NNRTI mutations K101E and V106 M were observed in plasma and CSF compartment, respectively. Two ART naïve participants had HIV-1 strain harbouring PI-associated DRMs in their CSF and not in plasma. One participant had M46MI mutation and the other had I84IT. Although there is discordance in these mutations between the CSF and plasma, according to the Stanford HIV drug resistance database, M46MI only confers resistance PI only when present with other mutation while I84T is not known to produce resistance to PI.^[26]^ These mutations occur at lower frequency in ART naïve participants but it was not possible to determine if their presence is due to transmitted drug resistance or the undisclosed ART status.^[26–28]^ If these participants were previously exposed to PIs then occurrence of these mutations solely in the CSF compartment would be attributable to low CNS penetration effectiveness of PIs via the BBB.^[6,29,30]^ In all these participants with HIV-1 DRM discordance, there was no evidence of viral escape as a result of present DRMs. One patient harboured HIV-1 strain with extensive DRMs and high HIV-1 VL in both CSF and plasma (patient ID:1736, Table 3). Inability of the participant to achieve suppressed HIV-1 VL even after 110 months of ART could be attributable to poor ART adherence resulting in development of DRMs.

One of the limitations of this study is that most studies define viral discordance as HIV-1 VL ≥ 0.5 or 1 log_10_ copies/mL in CSF as compared to plasma, but we defined HIV-1 VL discordance as any CSF VL higher than plasma VL and this may have implications on the clinical significance of the results obtained. A lack of a control group, that is, HIV infected individuals without CM was a limiting factor because HIV-associated CM can lead to secondary escape which is not necessarily due to compartmentalization but presence of pathogenic neurological infection such as CM. Studies with ultra-deep sequencing are warranted for the detection of HIV minority variants in the 2 compartments which may be missed by population sequencing. Despite the limitations, this is the first known study in Botswana to report on HIV-1C genetic discordance in CSF and plasma compartments of participants with HIV-associated CM.

In conclusion, low rates of CSF viral escape were observed in participants with HIV-associated CM in Botswana. We observed discordant DRMs between compartments. Further evaluation of HIV-1 viral factors that mediate CSF viral escape and HIV-1 DRM discordance may be important in understanding clinical implications in participants with HIV-associated CM.

## Acknowledgments

We thank greatly the study participants and AMBITION-cm study team.

NK, SG, SM, IK, TSH, JNJ, KKS and RM conceived and designed the experiments. NK, LE, DM, and MM conducted the experiments. NK, NOM, KM and SM analysed the results and compiled first draft. TSH and JNJ conceived original study (AMBITION-cm). TBL, DSL, KL, JNJ and TSH managed AMBITION-cm study. All authors reviewed and approved the final manuscript.

## Author contributions

**Conceptualization:** Nametso Kelentse, Sikhulile Moyo, Kaelo Seatla, Rosemary Musonda, Ishmael Kasvosve, Thomas S Harrison, Joseph N Jarvis, Simani Gaseitsiwe.

**Formal analysis:** Nametso Kelentse, Sikhulile Moyo, Natasha O Moraka, Kesaobaka Molebatsi.

**Funding acquisition:** Sikhulile Moyo, Rosemary Musonda, Simani Gaseitsiwe.

**Investigation:** Nametso Kelentse, Mompati Mogwele, Dorcas Maruapula, Lorato Esele.

**Methodology:** Nametso Kelentse, Sikhulile Moyo, Simani Gaseitsiwe.

**Project administration:** Nametso Kelentse.

**Resources:** Sikhulile Moyo, Kwana Lechiile, Dorcas Maruapula, Tshepo B Leeme, David S Lawrence, Thomas S Harrison, Joseph N Jarvis, Simani Gaseitsiwe.

**Supervision:** Rosemary Musonda, Ishmael Kasvosve, Simani Gaseitsiwe.

**Visualization:** Nametso Kelentse, Sikhulile Moyo, Simani Gaseitsiwe.

**Writing – original draft:** Nametso Kelentse, Sikhulile Moyo, Mompati Mogwele, Natasha O Moraka, Kaelo Seatla, Kesaobaka Molebatsi, Ishmael Kasvosve, Simani Gaseitsiwe.

**Writing – review & editing:** Nametso Kelentse, Sikhulile Moyo, Mompati Mogwele, Kwana Lechiile, Natasha O Moraka, Dorcas Maruapula, Kaelo Seatla, Lorato Esele, Kesaobaka Molebatsi, Tshepo B Leeme, David S Lawrence, Rosemary Musonda, Ishmael Kasvosve, Thomas S Harrison, Joseph N Jarvis, Simani Gaseitsiwe.

## Abbreviations

Abbreviations: AMBITION-cm = ambisome therapy induction optimisation for *Cryptococcal Meningitis*, aOR = adjusted odds ratio, ART = antiretroviral therapy, ARV = antiretroviral drugs, BBB = blood brain barrier, CM = cryptococcal meningitis, CNS = central nervous system, CSF = cerebrospinal fluid, DRM = drug resistance mutations, HIV = human immunodeficiency virus, PCR = polymerase chain reaction, RNA = ribonucleic acid, RT = reverse transcriptase, VL = viral load.
